# Effect of dialysis and transplantation on myocardial repolarization parameters and P‐wave dispersion in chronic kidney disease

**DOI:** 10.1002/joa3.12511

**Published:** 2021-02-08

**Authors:** Murat Akcay, Ufuk Yıldırım

**Affiliations:** ^1^ Department of Cardiology Faculty of Medicine Ondokuz Mayis University Samsun Turkey

**Keywords:** chronic kidney disease, dialysis and transplantation, P‐wave dispersion, Tp‐e interval, Tp‐e/QT ratio

## Abstract

**Background:**

Chronic kidney disease (CKD) patients are at higher risk for cardiac arrhythmias. The risk of arrhythmia may change with different treatment modalities. We proposed to compare the effects of varied therapy methods on myocardial repolarization parameters (Tp‐e, QT, QTc intervals, Tp‐e/QT, Tp‐e/QTc ratios) and P‐wave dispersion (PWD) in patients with CKD.

**Methods:**

Three groups were formed from the patients aged between 18 and 65 years, as Group 1 consisting of CKD patients receiving hemodialysis (HD) three times a week, Group 2 consisting of predialysis CKD patients and Group 3 consisting of CKD patients who underwent successful transplantation. All patients’ basic demographic data, risk factors, and echocardiographic parameters were recorded, and electrocardiographic repolarization parameters and PWD were analyzed.

**Results:**

The PR, QT, and QTc intervals were significantly shorter in the transplantation group compared to the other groups (*P* = .020, *P* < .001, *P* = .035; respectively). Tp‐e interval, Tp‐e/QT, and Tp‐e/QTc ratios were significantly higher in the predialysis group compared to the other groups (*P* < .001, *P* < .001, *P* = .001; respectively), while there was no significant variation between the HD and transplantation groups (*P* > .05). PWD was significantly increased in the predialysis group compared to other two groups (*P* < .001), while no significant variation between the HD and transplantation groups was observed.

**Conclusion:**

We found that the Tp‐e interval, Tp‐e/QT, Tp‐e/QTc, and PWD were significantly higher in the predialysis CKD group, but the PR, QT, and QTc intervals were significantly shorter in the transplantation group compared to the other groups. The prognostic significance and prediction of these parameters in arrhythmic events in CKD patients requires further evaluation with long‐time follow‐up.

## Graphical abstract

The Tp‐e interval, Tp‐e/QT and Tp‐e/QTc ratios, P‐wave dispersion (PWD) were significantly higher in pre‐dialysis chronic kidney disease group, but PR, QT, QTc intervals were significantly shorter in transplantation group compare to other groups. Our results may suggest that the predialysis group is the most increased risk for the ventricular arrhythmias and the least increased risk for the transplant patients. Again, prolonged PWD may suggest that the predialysis group is the most increased risk for atrial fibrillation compare to other groups.

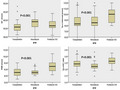

## 1 INTRODUCTION

Chronic kidney disease (CKD) is characterized by decreased glomerular filtration rate (GFR) (60 mL/min/1.73 m^2^) and/or the presence of renal damage, such as microalbuminuria, for more than 3 months.^1^ Cardiovascular diseases (CVD) are the major cause of morbidity and mortality in patients with CKD.^1^ Coronary artery disease, heart failure, pericarditis, atrial fibrillation (AF), and arrhythmias are the most common CVD causing death as a result of CKD.^1^, ^2^ Increased risk factors such as diabetes mellitus (DM), hypertension, hyperlipidemia, left ventricular hypertrophy, myocardial fibrosis, arterial stiffness, progression of atherosclerosis, and changes in the concentrations of serum electrolytes are the main contributing factors.^1^, ^2^

The risk of cardiovascular mortality and arrhythmias may be changed with different treatment modalities in patients with CKD.^2^ Several studies have demonstrated that hemodialysis (HD) affects a variety of electrolyte, fluid, and acid–base parameters, as well as electrocardiography (ECG) results.^3^ Some ECG changes are used as a predictive factor for arrhythmia. The peak to end interval of T wave (Tp‐e) is a measure of trans‐myocardial distribution of repolarization and may be related to dangerous rhythm irregularity and ventricular arrhythmias.^4^, ^5^ Again, raised Tp‐e/QT ratio is related to reentry mechanism for underlying pathophysiology in arrhythmic events.^6^, ^7^, ^8^ Myocardial repolarization parameters are evaluated with QT interval, corrected QT (QTc), Tp‐e interval, Tp‐e/QT, and Tp‐e/QTc ratio. The increase in these parameters is related with malignant rhythm disorders and sudden cardiac death.^7^, ^8^ P‐wave dispersion (PWD) defined as the prolongation of interatrial and intraatrial conduction duration as a result of irregular spread of sinus beats are well‐known predictors of AF and raised in patients with paroxysmal AF.^9^, ^10^, ^11^

In this study, we proposed to compare the effects of different treatment strategies on myocardial repolarization parameters and P‐wave dispersion in patients with CKD who received HD, patients with CKD in the predialysis period, and patients with CKD who underwent renal transplantation.

## 2 METHODS

### 2.1 Study population and study protocol

This study was a cross‐sectional and observational study. A total of 120 CKD patients followed‐up at the Nephrology Outpatient Clinic were directed to our clinic. Three groups were created from patients aged between 18 and 65 years, as Group 1 (40 patients) including CKD patients receiving HD three times a week, Group 2 (40 patients) consisting of predialysis CKD patients (GFR < 30 mL/min/1.73 m^2^) and Group 3 (40 patients) including CKD patients who underwent a successful transplantation (GFR > 60 mL/min/1.73 m^2^).

The exclusion parameters included a history of hypertrophic or restrictive cardiomyopathy, previous myocardial infarction, bypass operation history, low ejection fraction (EF < 50%), AF on ECG, QRS duration ≥120 msn, chronic pulmonary disease, severe liver disease, severe obesity (BMI ≥ 40 kg/m^2^), significant valvular disease or a history of valvular surgery, poor‐quality visualization on echocardiography, constrictive pericarditis, severe pericardial effusion, pregnancy, a history and/or findings of amyloidosis, electrolyte abnormalities (K, Na, Ca abnormalities) or receiving treatment because of the abnormality, and a history of kidney rejection. Again, patients with frequent palpitations or a detected paroxysmal AF attack were excluded. Informed consent was obtained from all patients.

All patients’ basic demographic data, age, height, weight, systolic and diastolic blood pressure, risk factors such as DM, hypertension and smoking, and CKD duration were questioned and recorded. The duration of dialysis and duration of renal transplantation were determined. Body mass index (BMI) was measured by dividing body weight in kilograms by the square of the height in meters (kg/m^2^). Laboratory parameters were evaluated in blood taken after 12 hours of fasting. Patients were routinely followed‐up by the department of nephrology, and no abnormalities were found in their electrolytes such as calcium, potassium, magnesium, and sodium. GFR was calculated using the Modification of Diet in Renal Disease (MDRD) formula. All data were collected on a day between dialysis days in the HD group.

### 2.2 Electrocardiography

Electrocardiography papers were recorded in a quiet room after 5 minutes of rest, with 20 mm/mV amplitude and 50 mm/s rate from an ECG machine (Cardiovit AT‐102 ECG, Schiller, Switzerland). All ECG papers were scanned and transferred to a computer. Adobe Photoshop software was used for 400% magnification of the parameters. Then all the parameters were analyzed by two blinded cardiologists. All twelve lead electrodes were analysed, but the V5 and DII leads were evaluated more detailed due to best reflects of apical‐basal and interventricular distribution of repolarization.^9^

The Tp‐e interval may be described as the distance from the highest amplitude of the T wave to its return to the isoelectric line. The QT interval was defined as the interval from the first deviation of the QRS wave to the end of the T wave. The R‐R interval was measured and used to compute the heart rate and to correct QT distance (QTc) with Bazett's formula (QT interval/√(RR interval)). The P‐wave times (Pmax and Pmin) were defined as the time from the beginning of the P‐wave to the end of the P‐wave according to the isoelectric line. All twelve lead electrodes were analyzed, and the difference among Pmax and Pmin was calculated and described as P‐wave dispersion (PWD = Pmax − Pmin). For each parameter, the ECG data was measured average three times.

Intraobserver and interobserver variability for myocardial repolarization parameters and P‐wave dispersion were assessed by repeating the measurements of 10 randomly selected individuals from each group. These parameters were reevaluated by the same physician at least 1 month later for intraobserver variability and by another one blinded to the data of the subjects for interobserver variability. Intraobserver and interobserver variability was lower than 5.0% and nonsignificant (*P* > .05) for all myocardial repolarization parameters and P‐wave dispersion.

### 2.3 Echocardiography

The echocardiographic evaluation was done in the left side decubitus position from standard acoustics views with Vivid E9 device (GE Medical System, Horten, Norway; 3.5‐MHz phased array transducer). Diameter of the left atrium, left ventricle end diastolic and end systolic dimensions, interventricular septum and posterior tissue diameter, and right ventricular diameter were measured in parasternal long‐axis view with two‐dimensional images. Ejection fraction was calculated using the modified Simpson method. M‐mode, two‐dimensional, Doppler and colored Doppler echocardiographic parameters of all patients were obtained and evaluated according to the American Society of Echocardiography standards.^12^ LV mass was measured using the Devereux's formula and indexed to body surface area (BSA). Left atrium (LA) volume was measured using the biplane area‐length technique and indexed to BSA.^12^

### 2.4 Statistical analysis

The data of patients were evaluated with IBM SPSS 20 (SPSS Inc, Chicago, IL, USA) program. In terms of variables suitability for normal distribution was analyzed using visual histograms and Shapiro‐Wilk test. Categorical variables were presented as numbers and percentages, continuous variables were presented as mean ± standard deviation if variables show normal distribution; and as median (minimum‐maximum) if variables show abnormal distribution. Chi‐square test was used to compare categorical parameters. For the parameters that were found unsuitable for normal distribution, the statistical method used was Kruskall‐Wallis *H* test and pairwise comparisons to detect statistically important changes among the three independent groups. One‐way ANOVA was used for the parameters with normal distribution to define important variations among the groups. Homogeneity of variances was determined with Levene's test. When variances were homogenous, Tukey's test was used for pairwise post‐hoc comparisons. When parameters were not homogenous, Tamhane's *T*_2_ test was used for pairwise post hoc comparisons. Intraobserver and interobserver variability were evaluated by coefficient of variation between the measurements. The statistically significant value was described as *P* < .05.

## 3 RESULTS

Demographic data for study in the three groups are summarized in Table 1. Patients in the transplantation group were younger, while there was no statistically important variation among the HD and predialysis groups. BMI values were lower in the HD group compared to the predialysis group, while no significant difference was found between the other groups. Blood pressure parameters were lower in the transplantation group compared to the predialysis group, while no significant variation was found compared to the HD group. Again, no statistically significant variation was found between the HD and predialysis groups. No statistically significant variation was found among the groups in terms of heart rate, DM, hypertension, and smoking risk factors (Table 1).

**TABLE 1:** Basic demographic values and risk factors for CKD patients according to the hemodialysis, predialysis and transplantation groups

Variable	Hemodialysis group (n = 40)	Predialysis CKD group (n = 40)	Transplantation group (n = 40)	*P* value
Age (y)	47.8 ± 8.8^b^	45.7 ± 9.7^b^	40 ± 9.1^a^	**0.001**
Gender				
Men, n (%)	18 (45)	15 (37.5)	23 (57.5)	0.194
Women, n (%)	22 (55)	25 (67.5)	17 (42.5)	
Height (cm)	161 ± 8.5^a^	161.6 ± 8.4^a^	164 ± 10^a^	0.481
Weight (kg)	63 ± 12.9^b^	71.7 ± 11.4^a^	70 ± 13.6^a^	**0.012**
BMI (kg/m^2^)	24.5 ± 4.9^a^	26.1 ± 4.6^b^	25.8 ± 4.2^ab^	**0.006**
Systolic BP (mmHg)	130 (90‐170)^ab^	135 (110‐180)^b^	120 (100‐180)^a^	**0.003**
Diastolic BP (mmHg)	80 (60‐105)^ab^	82.5 (70‐110)^b^	80 (60‐110)^a^	**0.021**
Heart rate (bpm/min)	75 (50‐116)	77 (50‐117)	77 (50‐109)	0.281
Diabetes mellitus, n (%)				
(−)	33 (82.5)	31 (77.5)	33 (82.5)	0.806
(+)	7 (17.5)	9 (22.5)	7 (17.5)	
Hypertension, n (%)				
(−)	8 (20)	8 (20)	8 (20)	1.0
(+)	32 (80)	32 (80)	32 (80)	
Cigarette, n (%)				
(−)	28 (70)	28 (70)	29 (72.5)	0.960
(+)	12 (30)	12 (30)	11 (27.5)	
CKD duration (y)	8.4 ± 5.8^b^	3 (1‐21)^a^	10 (3‐24)^b^	**<0.001**

Abbreviations: BMI, body mass index; BP, blood pressure; CKD, chronic kidney disease.

Bold values indicate statistically significant.

^a,b^ In pairwise comparisons between groups, there is no difference between groups with the same character.

Of the patients in the transplantation group, 82.5% (n = 33) received dialysis. Among these patients, 80% (n = 32) had living donors and 20% (n = 8) cadaver donors. The mean duration of dialysis before transplantation was found as 3 ± 4.3 years, and the mean time after transplantation was 3 ± 2.2 years. In the HD group, the mean duration of dialysis was 4.9 ± 4.5 years. In the predialysis group, duration of the diagnosis was found as minimum 1 and maximum 21 years. The median GFR value was calculated as 12 (6‐29) mg/dL/m^2^ in the predialysis group and 75.5 (61‐124) mg/dL/m^2^ in the transplantation group.

A statistically significantly lower level of hemoglobin was found in the HD group whereas a higher level of haemoglobin was observed in the transplantation group (*P* < .001). Left ventricular end diastolic diameter, posterior wall thickness were significantly lower in the transplantation group compared to both HD and predialysis groups (*P* < .001, *P* = .039). Again, left ventricular ejection fraction was significantly higher in the transplantation group compared to the other two groups. Left ventricular mass index, right ventricular size and systolic pulmonary artery pressure were also significantly lower in the transplantation group (*P* = .003, *P* = .023, *P* = .007; respectively). Again, left atrial anteroposterior diameter, left atrial volume and left atrial volume index were significantly lower in the transplantation group (*P* = .003, *P* = .023, *P* = .001; respectively) (Table 2).

**TABLE 2:** Basic echocardiographic and laboratory values of CKD patients according to the hemodialysis, predialysis and transplantation groups

Variable	Hemodialysis group (n = 40)	Predialysis CKD group (n = 40)	Transplantation group (n = 40)	*P* value
Hg (g/dL)	11.8 ± 1.6^b^	12 ± 2^a^	13.5 ± 1.6^c^	**<0.001**
BUN (mg/dL)	46.9 ± 19.8^b^	38.8 ± 24.5^b^	14.2 ± 3.9^a^	**<0.001**
Creatinine (mg/dL)	7.2 ± 3^c^	4.2 ± 3.2^b^	1.0 ± 0.2^a^	**<0.001**
Glucose (mg/dL)	87 (66‐176)	91 (69‐166)	91 (64‐182)	0.387
LVEDD (mm)	47.5 (33‐58)	46.9 ± 5.4	45 (36‐65)	0.155
LVESD (mm)	31.5 ± 5.2^b^	30.5 (23‐46)^b^	27 (19‐48)^a^	**<0.001**
IVS (mm)	13 (8‐17)	13 ± 2	12.5 (9‐17)	0.075
PW (mm)	11.5 ± 2.1^a^	11.6 (7‐16)^a^	11 (7‐15)^b^	**0.039**
LV mass index (g/m^2^)	134.8 ± 34.7^a^	125.4 ± 31.5^a^	105 ± 31.1^b^	**0.003**
LVEF %	61.5 ± 7.3^a^	65 (45‐75)^a^	71 ± 7^b^	**<0.001**
LA (a‐p) (mm)	37.3 ± 4.3^a^	36.3 ± 4.3^a^	34 ± 4.1^b^	**0.003**
LAV (mL)	46.2 ± 13.8^a^	44.2 ± 14.1^a^	39.2 ± 12.5^b^	**0.023**
LA volume index (mL/m^2^)	27.1 ± 7.7^a^	24.8 ± 8.4^a^	21.5 ± 7.1^b^	**0.001**
RV (mm)	25 ± 2^ab^	25 (23‐35)^b^	24.5 (18‐29)^a^	**0.023**
sPAP (mmHg)	30.6 ± 5.8^ab^	31.3 ± 7^b^	28 ± 6.6^a^	**0.007**

Abbreviations: EF, ejection fraction; Hg, haemoglobin; IVS, interventricular septum; LA (a‐p), left atrium‐parasternal long axis anteroposterior diameter; LAV, left atrial volume; LAVI, left atrial volume index; LVEDD, left ventricular end diastolic diameter; LVESD, left ventricular end systolic diameter; PW, posterior wall; RV, right ventricle; sPAP, systolic pulmonary artery pressure.

Bold values indicate statistically significant.

^a‐c^ In pairwise comparisons between groups, there is no difference between groups with the same character.

**FIGURE 1:**
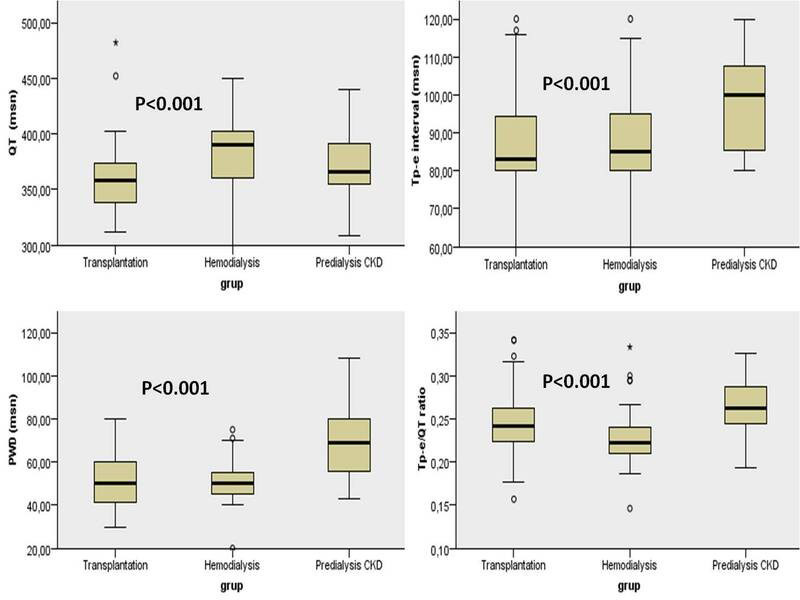
Imaging of distribution of the repolarization parameters and PWD according to the hemodialysis, predialysis and transplantation groups

In the evaluation of electrocardiographic parameters; PR interval, QT and QTc durations were significantly shorter in the transplantation group compared to the other groups (*P* = .020, *P* < .001, *P* = .035; respectively). While Tp‐e interval, Tp‐e/QT and Tp‐e/QTc ratios were significantly higher in the predialysis group compared to the other groups (*P* < .001, *P* < .001, *P* = .001; respectively), no significant variation was observed among the HD and transplantation groups (*P* > .05) (Table 3; Figure 1). The Pmax was significantly lower in the transplantation group compared to the predialysis group (*P* = .009), and no significant variation was observed between the other groups (*P* > .05). The Pmin was significantly lower in the predialysis group compared to the HD group (*P* = .001), and no significant variation was observed among the other groups (*P* > .05). PWD was significantly increased in the predialysis group compared to the other two groups (*P* < .001), while no significant variation was found between the HD and transplantation groups (Table 3; Figure 1).

**TABLE 3:** Myocardial repolarization values and P‐wave dispersion of CKD patients according to the hemodialysis, predialysis and transplantation groups

Variable	Hemodialysis group (n = 40)	Predialysis CKD group (n = 40)	Transplantation group (n = 40)	*P* value
Heart rate (bpm/min)	75 (50‐116)	77 (50‐117)	77 (50‐109)	0.281
PR interval (msn)	160 (100‐200)^b^	159 (112‐205)^b^	145.5 (104‐205)^a^	**0.020**
QRS interval (msn)	84 (65‐112)	84.5 (68‐110)	84.5 (68‐106)	0.970
QT interval (msn)	384 (300‐450)^b^	368 (304‐440)^b^	358 (258‐482)^a^	**<0.001**
QTc interval (msn)	421 (375‐524)^b^	423.5 (362‐514)^b^	413.5 (338‐536)^a^	**0.035**
Tp‐e interval (msn)	85 (60‐120)^a^	100 (60‐120)^b^	82 (60‐120)^a^	**<0.001**
Tp‐e/QT ratio	0.23 (0.14‐0.33)^a^	0.26 (0.20‐0.32)^b^	0.24 (0.15‐0.34)^a^	**<0.001**
Tp‐e/QTc ratio	0.21 (0.13‐0.29)^a^	0.23 (0.18‐0.29)^b^	0.21 (0.14‐0.35)^a^	**0.001**
Pmax. (msn)	120 (80‐140)^ab^	122 (90‐165)^b^	120 (80‐145)^a^	**0.009**
Pmin. (msn)	70 (45‐95)^a^	60 (45‐85)^b^	65 (43‐80)^ab^	**0.001**
PWD (msn)	50 (20‐75)^a^	65 (40‐108)^b^	50 (30‐80)^a^	**<0.001**

Abbreviation: PWD, P‐wave dispersion.

Bold values indicate statistically significant.

^a,b^ In pairwise comparisons between groups, there is no difference between groups with the same character.

## 4 DISCUSSION

In our study, we compared the Tp‐e interval, Tp‐e/QT ratio, Tp‐e/QTc ratio, QT, QTc interval, and P‐wave dispersion between the different treatment strategies in CKD patients. To our knowledge, there is no previous direct research compared among their three groups and we found that the PR, QT and QTc durations were significantly shorter in the transplantation group compared to the predialysis and HD groups. Furthermore, the Tp‐e interval, Tp‐e/QT, Tp‐e/QTc ratio, and PWD were significantly higher in the predialysis CKD group compared to the transplantation and HD groups.

Chronic kidney disease patients are known to be at higher risk for CVD and also lethal arrhythmias and sudden cardiac death.^1^, ^3^, ^13^ Furthermore, CVD are related to poor clinical endpoints in CKD patients. This pathology is defined as cardio renal syndrome in which dysfunction of one organ may trigger the dysfunction of other organs.^13^ Cardiovascular abnormalities are commonly evaluated by echocardiography and ECG. Echocardiography relies on the accessibility of experienced personals, but ECG is a simple, easy, and cheap noninvasive diagnostic and prognostic method for detection of structural and functional variations in the heart.^13^ Early diagnosis in CKD patients will benefit from aggressive cardio‐protective therapies.

The rate of cardiovascular morbidity and mortality varies with different treatment approaches in CKD. Left ventricular hypertrophy is the most encountered cardiac pathology including predialysis and posttransplantation periods in patients with CKD.^14^, ^15^, ^16^ In our study, although Interventricular septum (IVS) was hypertrophic in all treatment groups, PW was significantly less hypertrophied in the transplantation group. The reduced left ventricular ejection fraction and increased LA size have also been noticed to be common between the CKD patients.^13^, ^14^, ^15^, ^16^ In our study, Left ventricular ejection fraction (LVEF) was significantly higher and left atrium diameter was significantly lower in the transplantation group compared to the HD and predialysis groups.

The ECG changes show the electrophysiological properties of the myocardium and reflect a combination of dysregulation in ionic currents, metabolic changes, changes in serum electrolytes especially dyskalemia, dysmagnesemia, and secondary effects of drugs in CKD patients.^1^, ^17^ Both supraventricular and ventricular arrhythmias are common in CKD patients receiving HD therapy.^18^ Because of rapid changes in volume, electrolyte concentrations and metabolic abnormalities after dialysis, some ECG changes, significant arrhythmias, or sudden cardiac death may be seen.^3^ Prolongation of QRS and QTc interval should be kept in mind after HD and evaluated carefully in CKD patients.^3^ The study of Deo et al^17^ included 3587 CKD participants and demonstrated that increased heart rate, prolongation of PR and QTc intervals, and increased QRS duration were independent risk factors for cardiovascular death. Kollu et al^2^ showed that PWD, QTc dispersion, Tp‐e interval, and Tp‐e/QTc ratio increased with CKD stages 3‐5 compared with healthy controls. Sherif et al^19^ showed that retrospective analysis of 154 patients, the advancement of CKD resulted the important prolongation of QTc interval, independent of age, gender, and electrolyte abnormalities. Our results also support those of previous studies. The shorter of PR interval, QT and QTc intervals in the transplantation group and higher of the Tp‐e interval, Tp‐e/QT, Tp‐e/QTc ratios and PWD in the pre‐dialysis CKD group, might be explained with the effect of accumulating toxic, metabolic agents in the body.

Waks et al^6^ evaluated studies in CKD patients under HD, although small studies are promising, it is unclear whether ECG changes in CKD are mechanically associated with sudden cardiac death or only electrophysiological signs of more progressed CVD such as severe fibrosis or left ventricular hypertrophy. In the retrospective analysis of 205 CKD patients undergoing haemodialysis by Saour et al^20^ detected no association with prolongation of Tp‐e or Tp‐e/QT on dialysis with sudden cardiac death or all‐cause mortality.

Again, in our study, PWD was significantly increased in the predialysis group (*P* < .001). PWD was detected in 50 ms in the HD and transplantation group and 65 ms in the predialysis group. Huang et al^21^ showed that an increase in PWD and maximum P‐wave time were related with progression to dialysis or death and a fast decrease in renal function in predialysis CKD patients. But contradictory to our work, Tezcan et al^22^ detected a significant increase in Pmax time and PWD after HD compared to the predialysis period.

The risk of cardiovascular death is decreased after renal transplantation compared with those on dialysis, but still significantly higher than the general population especially in first year after the transplantation.^23^ The study of Amoozgar et al^24^ showed that in children with CKD, ventricular repolarization parameters recover after transplantation, although they still stay longer than the normal values. Monfared et al^25^ showed that renal transplantation decreased maximum QTc interval compared to predialysis and post‐HD that may be related to normalization of electrolytes and uremic condition.

This study is important, because the different effects of predialysis, HD and renal transplantation were demonstrated by changes in electrocardiographic parameters in CKD patients. Our study also supported the outcomes of most previous clinical researches. In the literature, there was no study demonstrating the relationship between the myocardial repolarization values and different treatment strategies in CKD. In our study, Tp‐e interval, Tp‐e/QT, Tp‐e/QTc ratio, and PWD were significantly higher in the predialysis CKD group compared with the transplantation and HD groups. Also, these results were independent of left ventricular mass index, left atrial volume and left atrial volume index.

### 4.1 Study limitations

Our study did not have a large patient population. We measured the electrocardiographic parameters from computer with magnified images. But this measurement technique has been performed and accepted in many previous studies. We evaluated the different treatment approaches on predictors of arrhythmias, such as PWD, QTc, Tp‐e, and Tp‐e/QT in CKD patients, but we could not perform a long‐term ambulatory cardiac rhythm analysis and follow the arrhythmic events. Also, possible effects of immunosuppressive drugs used by the patients in the transplantation group were not considered. Another limitation of our study is that ambulatory electrocardiographic monitoring cannot applied to detect paroxysmal AF attack and evaluate the dynamic change of repolarization parameters.

## 5 CONCLUSION

Myocardial repolarization parameters (Tp‐e interval, Tp‐e/QT and Tp‐e/QTc ratio) and P‐wave dispersion increased in predialysis CKD patients compared with transplantation and HD patients. However, the PR, QT, and QTc intervals were significantly shorter in the transplantation group compared to other groups. Our results may suggest that the predialysis group is at most increased risk for the ventricular arrhythmias and least increased risk for the transplant patients. The prognostic significance and prediction of these parameters in arrhythmic events in CKD patients requires further evaluation with long‐term follow‐up and large‐scale prospective studies.

## CONFLICT OF INTEREST

None declared.

## STATEMENT OF ETHICS

This study was approved by the Institutional Ethics Committee. The study was performed in accordance with the Declaration of Helsinki.

## References

[1] Boriani G , Savelieva I , Dan GA , Deharo JC , Ferro C , Israel CW , et al. Document reviewers. Chronic kidney disease in patients with cardiac rhythm disturbances or implantable electrical devices: clinical significance and implications for decision making‐a position paper of the European Heart Rhythm Association endorsed by the Heart Rhythm Society and the Asia Pacific Heart Rhythm Society. Europace. 2015;17(8):1169–96. 26108808 10.1093/europace/euv202 PMC6281310

[2] Kollu K , Altintepe L , Duran C , Topal M , Ecirli S . The assessment of P‐wave dispersion and myocardial repolarization parameters in patients with chronic kidney disease. Ren Fail. 2018;40(1):1–7. 29285964 10.1080/0886022X.2017.1419962 PMC6014377

[3] Astan R , Akpinar I , Karan A , et al. The effect of hemodialysis on electrocardiographic parameters. Ann Noninvasive Electrocardiol. 2015;20(3):253–7. 25201342 10.1111/anec.12209 PMC6931820

[4] Patel C , Burke JF , Patel H , et al. Is there a significant transmural gradient in repolarization time in the intact heart? Cellular basis of the T wave: a century of controversy. Circ Arrhythm Electrophysiol. 2009;2(1):80–8. 19808446 10.1161/CIRCEP.108.791830 PMC2662714

[5] Letsas KP , Charalampous C , Korantzopoulos P , et al. Novel indexes of heterogeneity of ventricular repolarization in subjects with early repolarization pattern. Europace. 2012;14(6):877–81. 22186777 10.1093/europace/eur390

[6] Waks JW , Tereshchenko LG , Parekh RS . Electrocardiographic predictors of mortality and sudden cardiac death in patients with end stage renal disease on hemodialysis. J Electrocardiol. 2016;49(6):848–54. 27554424 10.1016/j.jelectrocard.2016.07.020 PMC5159311

[7] Tse G , Yan BP . Traditional and novel electrocardiographic conduction and repolarization markers of sudden cardiac death. Europace. 2017;19(5):712–21. 27702850 10.1093/europace/euw280

[8] Gupta P , Patel C , Patel H , et al. T(p‐e)/QT ratio as an index of arrhythmogenesis. J Electrocardiol. 2008;41:567–74. 18790499 10.1016/j.jelectrocard.2008.07.016

[9] Pérez‐Riera AR , de Abreu LC , Barbosa‐Barros R , Grindler J , Fernandes‐Cardoso A , Baranchuk A . P‐wave dispersion: an update. Indian Pacing Electrophysiol J. 2016;16(4):126–33. 27924760 10.1016/j.ipej.2016.10.002 PMC5197451

[10] Dilaveris PE , Gialafos JE . P‐wave dispersion: a novel predictor of paroxysmal atrial fibrillation. Ann Noninvasive Electrocardiol. 2001;6(2):159–65. 11333174 10.1111/j.1542-474X.2001.tb00101.x PMC7027606

[11] Akcay M . The effect of moderate altitude on Tp‐e interval, Tp‐e/QT, QT, cQT and P‐wave dispersion. J Electrocardiol. 2018;51(6):929–33. 30497749 10.1016/j.jelectrocard.2018.07.016

[12] Lang RM , Bierig M , Devereux RB , et al. Chamber Quantification Writing Group, American Society of Echocardiography’s Guidelines and Standards Committee, European Association of Echocardiography. J Am Soc Echocardiogr. 2005;18:1440–63. 16376782 10.1016/j.echo.2005.10.005

[13] Chen SC , Huang JC , Su HM , Chiu YW , Chang JM , Hwang SJ , et al. Prognostic cardiovascular markers in chronic kidney disease. Kidney Blood Press Res. 2018;43(4):1388–407. 30153666 10.1159/000492953

[14] London GM . Left ventricular alterations and end‐stage renal disease. Nephrol Dial Transplant. 2002;17(Suppl 1):29–36. 10.1093/ndt/17.suppl_1.29 11812909

[15] Yildirim U , Gulel O , Eksi A , Dilek M , Demircan S , Sahin M . The effect of different treatment strategies on left ventricular myocardial deformation parameters in patients with chronic renal failure. Int J Cardiovasc Imaging. 2018;34(11):1731–9. 29948637 10.1007/s10554-018-1390-5

[16] Liu YW , Su CT , Huang YY , et al. Left ventricular systolic strain in chronic kidney disease and hemodialysis patients. Am J Nephrol. 2011;33:84–90. 21178338 10.1159/000322709

[17] Deo R , Shou H , Soliman EZ , et al. Electrocardiographic measures and prediction of cardiovascular and noncardiovascular death in CKD. J Am Soc Nephrol. 2016;27(2):559–69. 26160896 10.1681/ASN.2014101045 PMC4731112

[18] Bozbas H , Atar I , Yildirir A , et al. Prevalence and predictors of arrhythmia in end stage renal disease patients on hemodialysis. Ren Fail. 2007;29(3):331–9. 17497448 10.1080/08860220701191237

[19] Sherif KA , Abo‐Salem E , Panikkath R , Nusrat M , Tuncel M . Cardiac repolarization abnormalities among patients with various stages of chronic kidney disease. Clin Cardiol. 2014;37(7):417–21. 25043948 10.1002/clc.22277 PMC6649453

[20] Saour BM , Wang JH , Lavelle MP , et al. TpTe and TpTe/QT: novel markers to predict sudden cardiac death in ESRD? J Bras Nefrol. 2019;41(1):38–47. 30118535 10.1590/2175-8239-JBN-2017-0021 PMC6534015

[21] Huang JC , Wei SY , Chen SC , et al. P wave dispersion and maximum p wave duration are associated with renal outcomes in chronic kidney disease. PLoS One. 2014;9(7):e101962. 25006682 10.1371/journal.pone.0101962 PMC4090207

[22] Tezcan UK , Amasyali B , Can I , et al. Increased P wave dispersion and maximum P wave duration after hemodialysis. Ann Noninvasive Electrocardiol. 2004;9(1):34–8. 14731214 10.1111/j.1542-474X.2004.91529.x PMC6932169

[23] Mozos I . Laboratory markers of ventricular arrhythmia risk in renal failure. Biomed Res Int. 2014;2014:509204. 24982887 10.1155/2014/509204 PMC4058221

[24] Amoozgar H , Tavakoli A , Fallahzadeh MH , Derakhshan A , Basiratnia M . The effect of renal transplantation on ventricular repolarization in children with chronic renal failure. Int J Organ Transplant Med. 2013;4(4):144–9. 25013667 PMC4089322

[25] Monfared A , Atrkar Roshan Z , Salari A , Asadi F , Lebadi M , Khosravi M , et al. QT intervals in patients receiving a renal transplant. Exp Clin Transplant. 2012;10(2):105–9. 22432752 10.6002/ect.2011.0117

